# Whole-brain irradiation differentially modifies neurotransmitters levels and receptors in the hypothalamus and the prefrontal cortex

**DOI:** 10.1186/s13014-020-01716-y

**Published:** 2020-11-23

**Authors:** Javier Franco-Pérez, Sergio Montes, Josué Sánchez-Hernández, Paola Ballesteros-Zebadúa

**Affiliations:** 1 Laboratory of Physiology of Reticular Formation, National Institute of Neurology and Neurosurgery, INNN, Insurgentes Sur 3877, Col. La Fama, C.P. 14269 Mexico City, Mexico; 2 Laboratory of Neurochemistry, National Institute of Neurology and Neurosurgery, INNN, Insurgentes Sur 3877, Col. La Fama, C.P. 14269 Mexico City, Mexico; 3 Laboratory of Medical Physics, National Institute of Neurology and Neurosurgery, INNN, Insurgentes Sur 3877, Col. La Fama, C.P. 14269 Mexico City, Mexico

**Keywords:** Whole brain irradiation, Amino acids, Neurotransmitters, Prefrontal cortex, Hypothalamus, GABAa, GABAb, NR1, NR2A

## Abstract

**Background:**

Whole-brain radiotherapy is a primary treatment for brain tumors and brain metastasis, but it also induces long-term undesired effects. Since cognitive impairment can occur, research on the etiology of secondary effects has focused on the hippocampus. Often overlooked, the hypothalamus controls critical homeostatic functions, some of which are also susceptible after whole-brain radiotherapy. Therefore, using whole-brain irradiation (WBI) in a rat model, we measured neurotransmitters and receptors in the hypothalamus. The prefrontal cortex and brainstem were also analyzed since they are highly connected to the hypothalamus and its regulatory processes.

**Methods:**

Male Wistar rats were exposed to WBI with 11 Gy (Biologically Effective Dose = 72 Gy). After 1 month, we evaluated changes in gamma-aminobutyric acid (GABA), glycine, taurine, aspartate, glutamate, and glutamine in the hypothalamus, prefrontal cortex, and brainstem according to an HPLC method. Ratios of Glutamate/GABA and Glutamine/Glutamate were calculated. Through Western Blott analysis, we measured the expression of GABAa and GABAb receptors, and NR1 and NR2A subunits of NMDA receptors. Changes were analyzed comparing results with sham controls using the non-parametric Mann–Whitney U test (*p* < 0.05).

**Results:**

WBI with 11 Gy induced significantly lower levels of GABA, glycine, taurine, aspartate, and GABAa receptor in the hypothalamus. Also, in the hypothalamus, a higher Glutamate/GABA ratio was found after irradiation. In the prefrontal cortex, WBI induced significant increases of glutamine and glutamate, Glutamine/Glutamate ratio, and increased expression of both GABAa receptor and NMDA receptor NR1 subunit. The brainstem showed no statistically significant changes after irradiation.

**Conclusion:**

Our findings confirm that WBI can affect rat brain regions differently and opens new avenues for study. After 1 month, WBI decreases inhibitory neurotransmitters and receptors in the hypothalamus and, conversely, increases excitatory neurotransmitters and receptors in the prefrontal cortex. Increments in Glutamate/GABA in the hypothalamus and Glutamine/Glutamate in the frontal cortex indicate a neurochemical imbalance. Found changes could be related to several reported radiotherapy secondary effects, suggesting new prospects for therapeutic targets.

## Background

Whole-brain radiotherapy is a primary medical treatment for some types of brain cancer, especially for brain metastasis. Although it improves patient’s survival, it is irrefutable that its use has been associated with several adverse effects. According to the time of appearance, these effects have been classified as acute (hours to days), early-delayed (1–6 months), and late-delayed (more than 6 months after) [1]. It is currently well established that whole-brain irradiation (WBI) at therapeutic doses leads to an increased risk for late-delayed cognitive impairments [2]. The hippocampus is the main structure associated with memory and cognition, and thus the most studied after brain irradiation. Authors have proposed that the ablation of hippocampal neurogenesis plays a crucial role in cognitive impairment after radiation [3, 4]. It has also been suggested that the cellular mechanism underlying cognitive deficit involves alterations in receptors related to synaptic plasticity. Therefore, using in vitro and in vivo models, it has been shown that irradiation changes the expression of excitatory and inhibitory receptors and neurotransmitters in the hippocampus [5–9].

WBI induces a complex initial cascade of neurochemical processes, which can trigger the appearance of early-delayed effects often considered transient and clinically overlooked. Early-delayed effects include Radiation Somnolence Syndrome (RSS) characterized by increased sleep during the day, fatigue, decreased appetite, and weight loss [10]. Indeed, fatigue, loss of appetite, and weakness are the most frequent symptoms reported to worsen in patients after WBI [11]. Many of these symptoms are closely related to alterations of the hypothalamic function. Moreover, it has been observed that endocrine disruption of the hypothalamic–pituitary–adrenal (HPA) axis can frequently appear after WBI [12]. The hypothalamus is a region that controls many critical homeostatic functions, including those that are perturbed after whole-brain radiotherapy [12, 13]. Therefore, it is relevant to analyze hypothalamic neurochemical changes after WBI. Consequently, in this work, we measured excitatory and inhibitory neurotransmitters levels in the hypothalamus. Additionally, we measured the expression of gamma-aminobutyric acid (GABA) receptors (GABAa and GABAb) and NMDA receptor subunits NR1 and NR2A since they have previously shown variations after different irradiation doses in other brain structures [5, 6, 14]. Moreover, we calculated Glutamate/GABA and Glutamine/Glutamate ratios as they have been used as markers of neurochemical brain balance [15, 16].

The brain response to radiation fluctuates according to the analyzed region. Thus, Todorovic et al. [17] demonstrated that the antioxidant response after radiation is lower in the hippocampus than the cerebral cortex. For this reason, we also analyze regions such as the prefrontal cortex and brainstem. Both structures are highly connected to the hypothalamus and are considered critical for cognitive processes, sleep regulation, and neuroimmune stress response [18, 19].

The data presented here demonstrate that WBI produces early delayed effects in the expression of receptors and neurotransmitters levels at the hypothalamus and prefrontal cortex. Its possible implications for future research are discussed.

## Methods

### Animals

Twenty-four male Wistar rats (270–300 g) were used. Animals were housed in a room with controlled temperature (22 ± 2 °C), light–dark cycle (12:12), and ad libitum access to water and food. All animals were handled according to Mexican Official Norms for the production, care, and use of laboratory animals (NOM-062-Z00-1999). Additionally, the Guide for the Care and Use of Laboratory Animals (NIH Guide) was revised and used as guidelines.

### Whole-brain irradiation

The irradiation was performed using a micro-multileaf collimator coupled to a linear accelerator for head treatments (Novalis Varian, 6 MV), as previously reported [20]. A prescription dose of 11 Gy was chosen since it corresponds to a BED (Biologically effective dose) of 72 Gy similar to the one used clinically (10 Fx, 3 Gy α/β = 2 Gy). All animals were deeply anesthetized by administering a mixture of ketamine (100 mg/kg) as a sedative agent, and xylazine (10 mg/kg) as a muscle relaxant. Once the animals showed sensory stimulation response inhibition, they were immobilized in a custom device and fixed to the treatment table. A single dose of the drugs was sufficient to carry out the WBI. The dynamic arcs technique was employed at a dose rate of 500 UM/min. As with patients, homogenous coverage was achieved all over the brain (RTOG homogeneity index HI = 1.3) while brain surrounding structures were protected. Sham animals were mounted but received no dose. We used rat CT images and the software iPlan (BrainLab Germany) for the treatment planning, and the dose verification was performed using Monte Carlo techniques [20].

### Brain amino acids analysis

Rats were killed by decapitation 1 month after the whole brain irradiation or sham manipulation. All the animals were sacrificed at the light phase between 9:00 and 10:00 a.m. to avoid circadian variations. Brain regions dissection was based on previously published protocols [21, 22]. Briefly, after decapitation, we extracted the brain by opening the skull through the midline. To obtain the prefrontal cortex, we first separate the olfactory bulb. Later, with the help of a rat brain slicer matrix, we made a 1-mm coronal cut to discard mainly the motor cortex, then we obtained another 1-mm coronal cut. In this slice, we took the genus corpus callosum and the capsula externa as a reference to delimitate and separate the striatum and thus get the prefrontal cortex sample. After that, we placed the brain ventral side up and located the optical chiasm and the midbrain in the anterior and posterior parts. With the help of a spatula, we punctured around such anatomical structures, and thus we obtained the hypothalamus. Finally, the brainstem was dissected after the elimination of the cerebellum and the inferior and superior colliculus. The brainstem portion included the medulla oblongata and pons. The brain regions were quickly dissected using the illustrations and coordinates shown in Paxinos and Watson Atlas [23]. The samples were stored at − 70 °C until later analysis. The aspartate (Asp), GABA, glutamate (Glu), glutamine (Gln), glycine (Gly), and taurine (Tau) contents were determined according to a method previously reported [24]. Briefly, tissue was homogenized and centrifuged at 4000×*g* for 10 min at 4 °C, and the supernatants were kept at − 70 °C until assayed. The amino acid content was analyzed using a high-performance liquid chromatography (HPLC) system Agilent 1100 series (Agilent Technologies) equipped with a fluorescence detector and Adsorbosphere ortho-phthalaldehyde (OPA) column (Alltech). The mobile phase consisted of a 50-mM sodium acetate buffer (pH 5.9) solution containing 1.5% tetrahydrofuran and HPLC-grade methanol. The pre-column derivatization procedure was carried out by mixing 100 µL of sample and 100 µL of OPA reagent. The content was expressed as micromole of amino acid per gram of wet tissue (mean ± SEM).

### Brain receptors analysis

The expression of GABAa and GABAb receptors, and NR1 and NR2A subunits of NMDA receptors was quantified by Western-Blott according to a method previously reported [25]. Briefly, the frozen samples from the hypothalamus, prefrontal cortex, and brainstem were homogenized in RIPA buffer containing a cocktail of protease inhibitors (Sigma Aldrich, USA). Homogenates were centrifuged at 10,000×*g* for 10 min at 4 °C, and the supernatants recovered. Protein concentration was measured using the BCA method. An aliquot was mixed with Laemmli sample buffer and denatured at 100 °C for 5 min. Polyacrylamide gels (10%) were loaded with 50 µg of protein. Proteins were transferred onto nitrocellulose membranes (Bio-Rad, USA) and then blocked with 5% nonfat dry milk diluted in TBST for one h at room temperature. Next, membranes were incubated overnight at 4 °C with either anti-GABAb mouse monoclonal antibody (1:1000, SC-166408, Santa Cruz, USA), anti-GABAa mouse monoclonal antibody (1:1000, sc-376282, Santa Cruz, USA), anti-NR1 monoclonal mouse antibody (1:200, BML-SA493-0015, Enzo, USA) or anti-NR2A monoclonal mouse antibody (1:200, sc-31540, Santa Cruz, USA) diluted in TBST containing 5% nonfat dry milk. After washing with TBST, membranes were incubated for 1 h at room temperature with anti-mouse IgG HRP conjugated (1:10000, SAB3701105-2, Sigma Aldrich, USA). Protein bands were observed by chemiluminescence using Luminata Forte (Millipore, USA) and an imaging system Fusion Solo S (Vilber, France). After detection, we submerged membranes in stripping buffer washed with TBST, blocked with TBST containing 5% nonfat dry milk, and incubated with anti-α Tubulin monoclonal mouse antibody (1:1000, sc23948, Santa Cruz, USA) as protein loading reference.

### Statistical analysis

We compared sham manipulation data with irradiated animals running the two independent samples non-parametric Mann–Whitney U test in SPSS (v 20 IBM). Differences were considered significant if *p* ≤ 0.05.

## Results

**Fig. 1:**
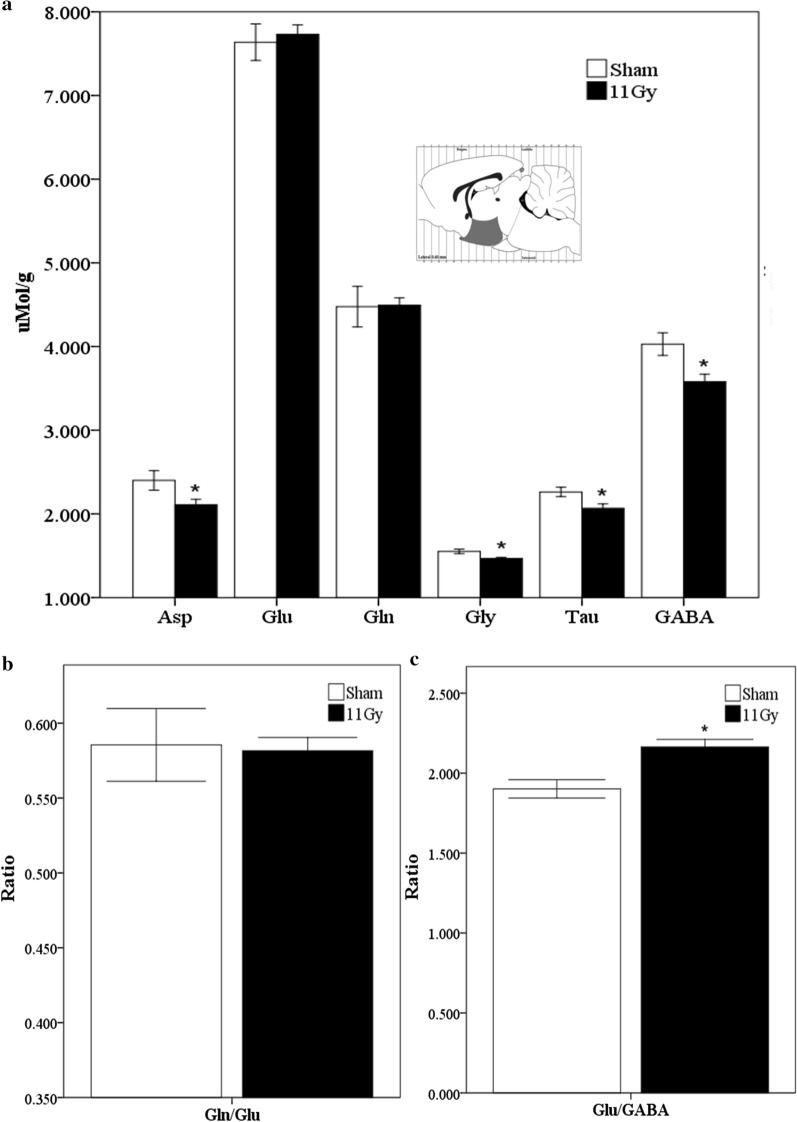
Neurotransmitters analysis in the hypothalamus 1 month after sham (white bars) or treatment with 11 Gy whole-brain irradiation (black bars). The analyzed region is indicated in a Paxinos and Watson diagram [21]. **a** The concentration (μMol/g of fresh tissue) of the following amino acids: Asp, Glu, Gln, Gly, Tau, and GABA. Glutamine/Glutamate and Glutamate/GABA ratios are showed in **b**, **c**, respectively. Data are expressed as means ± SEM. Groups were statistically compared using the Mann–Whitney U test *p ≤ 0.05

In the present study, Wistar rats were treated with a WBI protocol calculated to guarantee adjacent tissue protection, especially the mucosal tract. Consequently, no adverse peripheral reactions were observed. The HPLC method was modified to detect and measure six amino acid neurotransmitters in the same chromatogram. Figure 1a indicates that 1 month after WBI, there was a significant decrease in GABA (*p* = 0.015), the main inhibitory neurotransmitter in the hypothalamus. The levels of other inhibitory neurotransmitters such as Gly and Tau also decreased significantly (*p* = 0.04) in the hypothalamus compared to those detected in sham rats (Fig. 1a). Similarly, we observed a significant decrease of the excitatory amino acid Asp (*p* = 0.05) (Fig. 1a). In the hypothalamus, we also observed that the amino acids with the highest concentration were Glu > Gln > GABA. On the contrary, the Gly content was the lowest (Fig. 1a). We further examined the Gln/Glu and Glu/GABA ratios since the balance between inhibition and excitation is essential for the neurotransmission in the brain. Therefore, radiation did not induce significant changes in the Gln/Glu ratio (Fig. 1b); however, there was a significant increase of the Glu/GABA ratio in the hypothalamus after WBI (*p* = 0.004) (Fig. 1c).

**Fig. 2:**
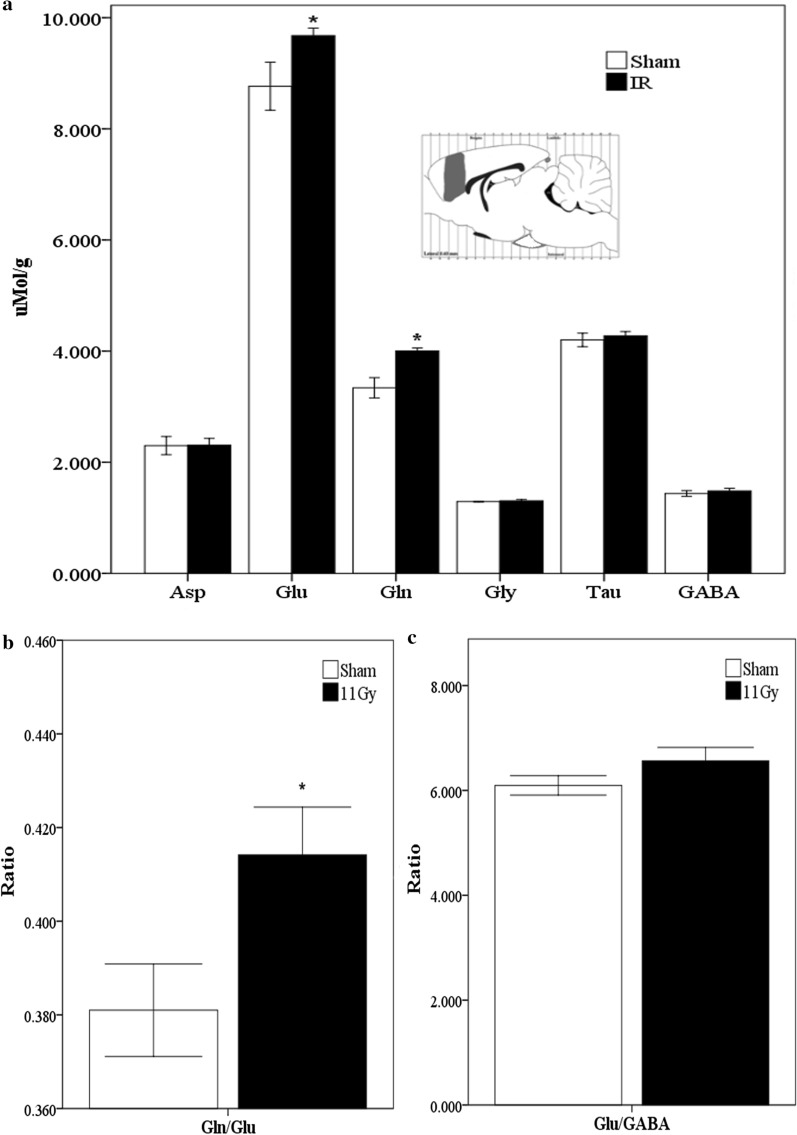
Neurotransmitter levels in the prefrontal cortex 1 month after sham (white bars) or treatment with 11 Gy whole-brain irradiation (black bars). The analyzed region is indicated in a Paxinos and Watson diagram [21]. **a** The concentration (μMol/g of fresh tissue) of the following amino acids: Asp, Glu, Gln, Gly, Tau, and GABA. Glutamine/Glutamate and Glutamate/GABA ratios are showed in **b**, **c**, respectively. Data are expressed as means ± SEM. Groups were statistically compared using the Mann–Whitney U test *p ≤ 0.05

By contrast, in the prefrontal cortex, an alteration of the glutamatergic transmission was observed, since excitatory neurotransmitters were increased 1 month after treatment. Figure 2a illustrates how levels of Glu and Gln were significantly increased (*p* = 0.04, *p* = 0.004, respectively).

Compared with the other structures analyzed, we found that the prefrontal cortex contains the highest levels of Glu and, on the contrary, the lowest levels of GABA (Fig. 2a). Figure 2b shows that WBI induced a significant increase in the Gln/Glu ratio (*p* = 0.04), suggesting that the metabolism of Glu could be disrupted in the prefrontal cortex of irradiated rats. Conversely, no differences were observed in the Glu/GABA ratio (Fig. 2c).

**Fig. 3:**
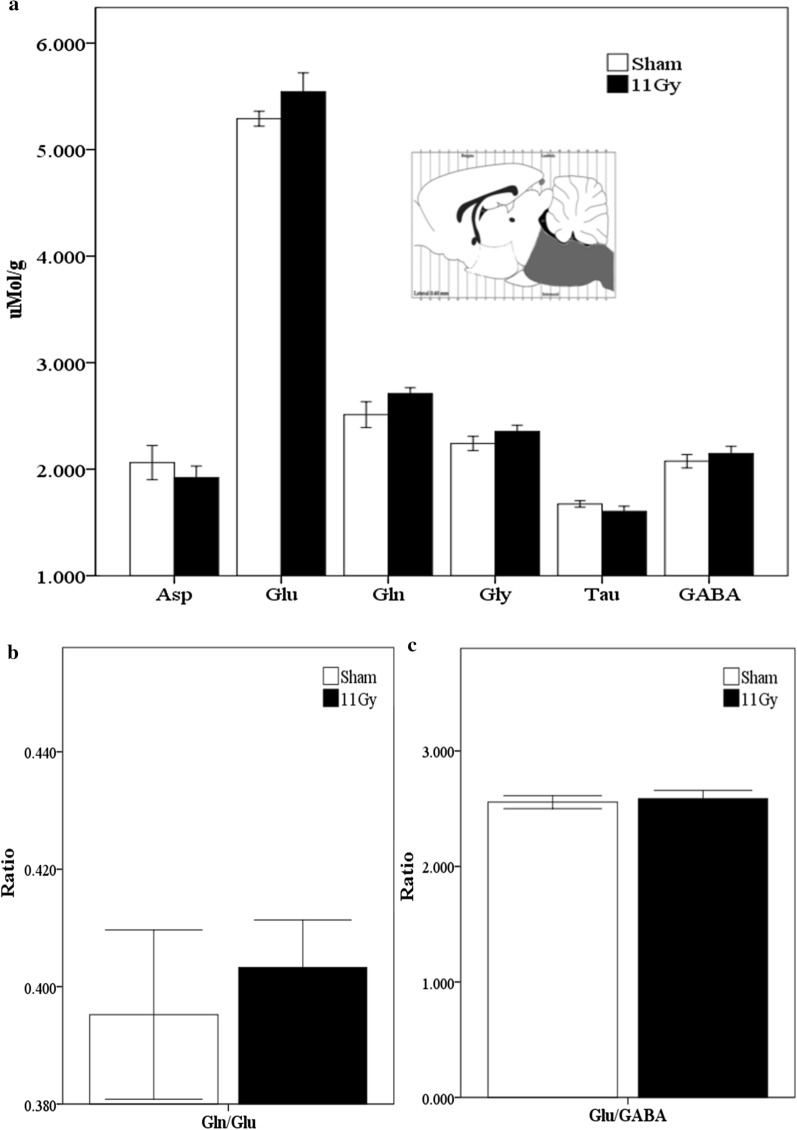
Neurotransmitters analysis in the brainstem 1 month after sham (white bars) or treatment with 11 Gy whole-brain irradiation (black bars). The analyzed region is indicated in a Paxinos and Watson diagram [21]. **a** The concentration (μMol/g of fresh tissue) of the following amino acids: Asp, Glu, Gln, Gly, Tau, and GABA. Glutamine/Glutamate and Glutamate/GABA ratios are showed in **b**, **c**, respectively. Data are expressed as means ± SEM. Groups were statistically compared using the Mann–Whitney U test *p ≤ 0.05. No statistical differences were found between groups

In the brainstem, we detected that the amino acids with the highest concentration were Glu > Gln > Gly. In contrast, Tau was the least abundant. After performing the statistical analysis, we noticed that both the amino acid levels and the ratios remained without significant changes (Fig. 3a–c).

**Fig. 4:**
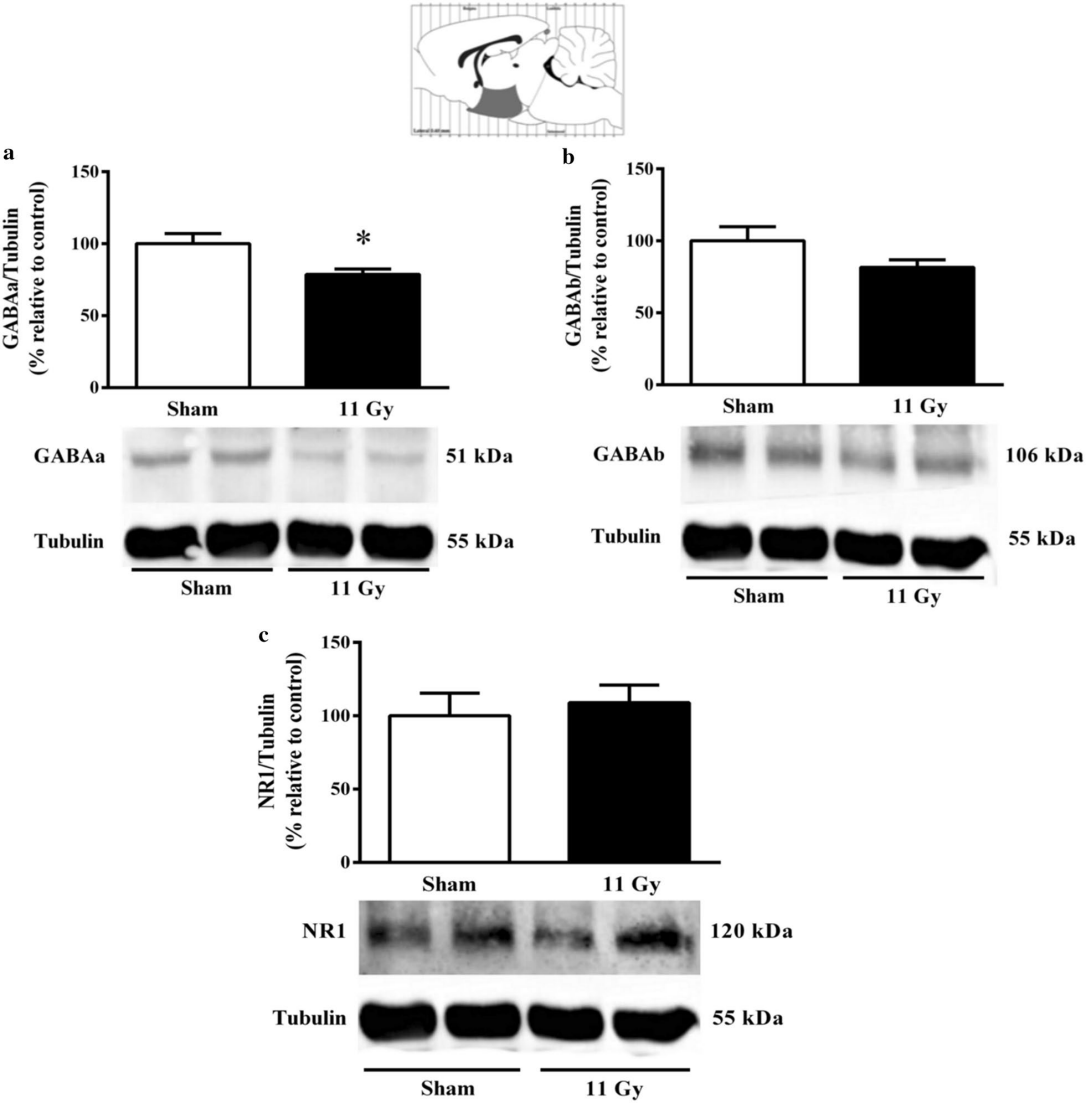
Expression of GABAa (**a**) GABAb (**b**), and NR1 subunit of the NMDA receptor (**c**). The protein expression was analyzed in the rat hypothalamus by Western Blott 1 month after sham (white bars) or whole-brain irradiation with a single dose of 11 Gy (black bars). The expression of the NR2A subunit was below standardized detection (data not shown). Data are expressed as means ± SEM. *p < 0.05 compared with the sham group using the Mann–Whitney U test

One month after treatment, WBI also induced changes in the protein expression of some inhibitory and excitatory receptors. In the hypothalamus, the expression of GABAa receptors was decreased significantly (*p* = 0.02) (Fig. 4a). A similar tendency was observed with GABAb; however, this effect was not significant (Fig. 4b). We analyzed different subunits of the NMDA receptor; nevertheless, no NR-1 changes were distinguished in irradiated rats (Fig. 4c). Detection of the NR2A subunit in the hypothalamus was shallow to raise comparisons, and we avoid further increasing the protein concentration to elude tubulin signal saturation.

**Fig. 5:**
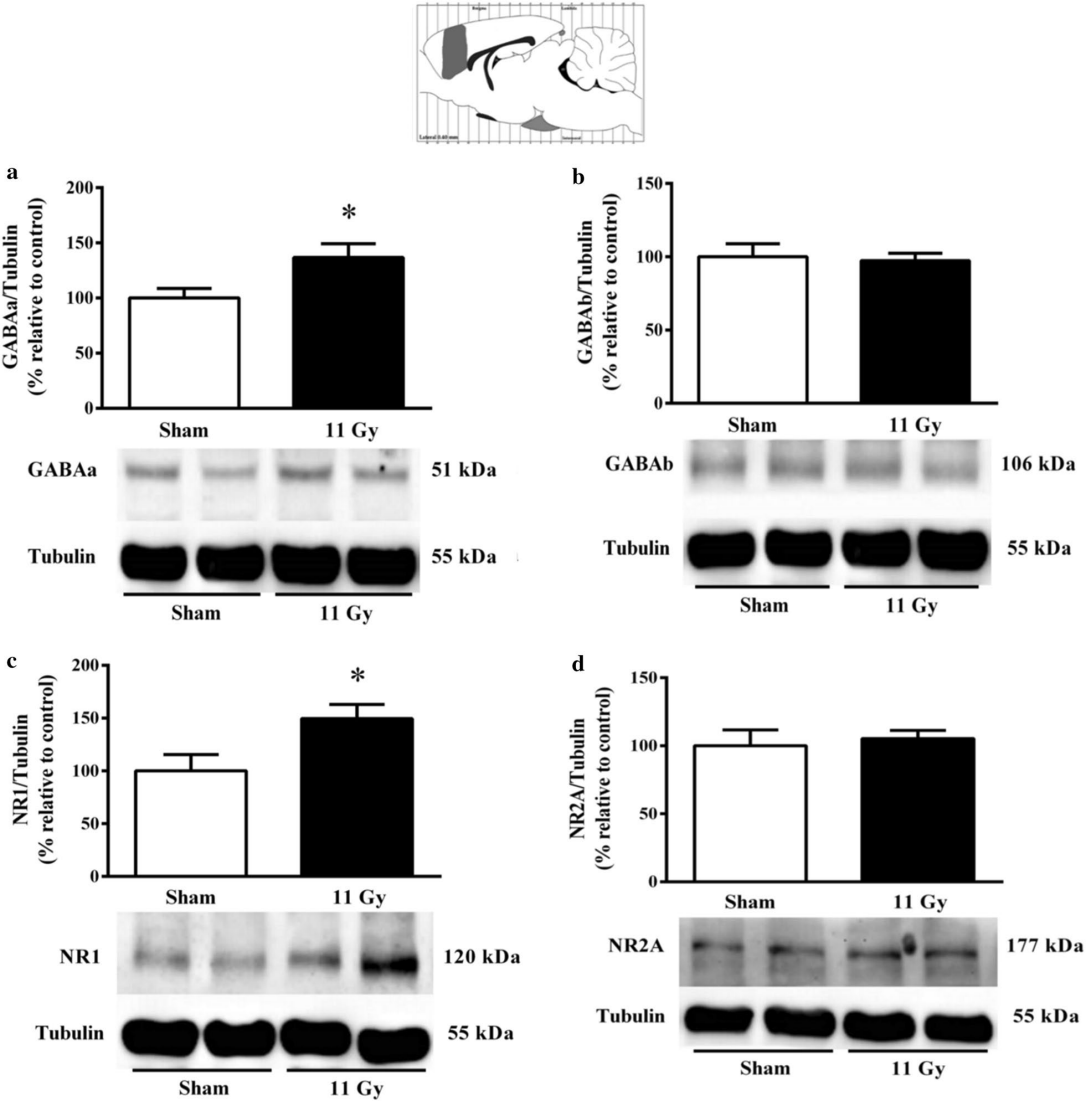
Expression of GABAa (**a**), GABAb (**b**), NR1 (**c**), and NR2A subunit of the NMDA receptor (**d**). The protein expression was analyzed in the rat prefrontal cortex by Western Blott 1 month after sham (white bars) or whole-brain irradiation with a single dose of 11 Gy (black bars). Data are expressed as means ± SEM. *p < 0.05 compared with the sham group using the Mann–Whitney U test

Mostly, the radiation upregulated the expression of some receptors in the prefrontal cortex. Specifically, the WBI with a single dose of 11 Gy induced a significant increase of the GABAa receptor (*p* = 0.05) (Fig. 5a) and NMDA receptor NR1 subunit (*p* = 0.05) (Fig. 5c). Conversely, no significant changes were detected when we analyzed the expression of GABAb (Fig. 5b) and the NMDA receptor NR2A subunit (Fig. 5d).

**Fig. 6:**
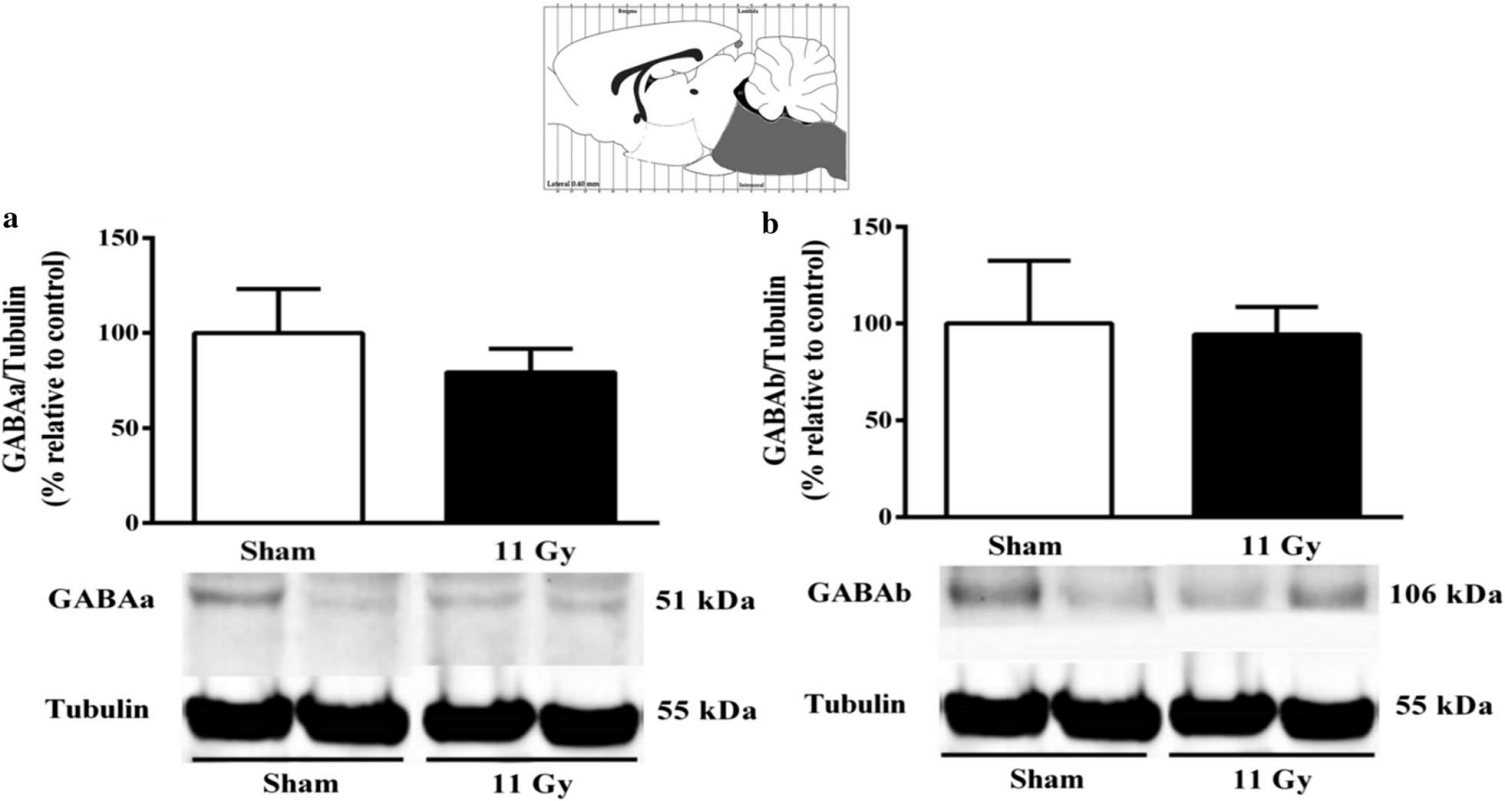
Expression of GABAa (**a**) and GABAb (**b**). The protein expression was analyzed in the brainstem by Western Blott 1 month after sham (white bars) or whole-brain irradiation with a single dose of 11 Gy (black bars). It was unable to detect any evidence of the NMDA subunits in the brainstem. Data are expressed as means ± SEM. *p < 0.05 compared with the sham group using the Mann–Whitney U test

Although we observed a decrease in the GABAa receptor in the brainstem, this was not significant (Fig. 6a). Likewise, the GABAb receptor showed no changes (Fig. 6b). We were unable to detect any evidence of expression of the NMDA subunits in the brainstem.

## Discussion

Here we used an irradiation rodent model using a single fraction (11 Gy) to resemble a biologically equivalent dose of 72 Gy that corresponds to a traditional scheme of whole-brain radiotherapy with 10 Fractions of 3 Gy using an α/β = 2 for the healthy tissue, according to the linear-quadratic model. Similar dose schemes in rodents have proof to efficiently replicate the behavioral effects observed in patients after whole brain radiotherapy, like somnolence and cognitive effects [20, 26–28]. Therefore, this dose radiation scheme is useful in analyzing the substantial impairments associated with whole-brain irradiation.

Our results indicate that WBI differentially modifies the levels of some neurotransmitters and receptors in the analyzed brain regions. GABA, the primary inhibitory neurotransmitter in the brain, was decreased in the hypothalamus 1 month after WBI. It is well known that inhibitory neurotransmission occurs through GABA interaction with two classes of receptors, ionotropic GABAa receptors and metabotropic GABAb receptors [29]. Remarkably, in this study, we showed that WBI also prompted a reduction in the expression of GABAa receptors in the hypothalamus. Studies have proposed that the regulation of the stress response carried out by the HPA axis involves the participation of GABAa receptors located in the hypothalamus [30]. Consistently, infant female rats irradiated with a lower dose (5 Gy) showed a reduction in the hypothalamic levels of GABA [30]. The same infant rats also exhibited increased levels of the gonadotropic releasing hormone (GnRH) and precocious puberty symptoms [31], supporting GABA’s relevance in the endocrine regulation of the HPA axis after cranial irradiation.

Likewise, low GABA levels in the hypothalamus are associated with fatigue, a symptom commonly reported after whole-brain radiotherapy [11]. Also, it has been suggested that the negative modulation of the GABAa function stimulates the occurrence of chronic fatigue syndrome [32]. Otherwise, fatigue has also been correlated with increased levels of inflammatory brain cytokines. Interestingly, we have previously reported high hypothalamic levels of inflammatory cytokine IL-1β after irradiation [20]. Therefore, correlations between inflammatory response and neurochemical changes occurring in the hypothalamus after brain irradiation should be further explored.

Moreover, hypothalamic GABA also participates in the regulation of feeding behavior [33]. For example, the administration of GABAa agonist muscimol into hypothalamic nuclei stimulates feeding. This response was inhibited by GABAa antagonist bicuculline [34]. Therefore, declining the GABAergic neurotransmission in the hypothalamus could be contributing to the decreased appetite and weight loss observed after WBI [11, 35–37]. Hypothalamic GABA neurons are also known to regulate several physiological and behavioral responses associated with anxiety and stress. Shekhar [38] showed that GABAergic activity inhibition in the dorsomedial hypothalamic area elicited evident signs of anxiety in rats. Therefore, we suggest that reduced GABA activity in the hypothalamus could be associated to anxiety behaviors observed after WBI [39].

GABA release in the posterior hypothalamus and GABAa activation have somnogenic effects [40]. Since previous results have shown that WBI may induce sleep, reduced expression in GABAa may seem contradictory [10, 20]. Nevertheless, we must remember that for evaluating neurotransmitters, animals were euthanized during the day, and incremented sleepiness has been previously observed during the dark phase. Still, further experiments could give more information about circadian variations in neurotransmitters in response to radiation and its correlation with sleep disturbances.

Interestingly, the observed reduction in GABA in the hypothalamus led to a significant increase in the Glu/GABA ratio, which points out the prevalence of excitatory processes. Increased Glu/GABA ratio may lead to neurotoxic effects as Glu/GABA ratio is commonly increased in other brain damage models like traumatic brain injury and kindling epilepsy [15, 41].

Glycine is an inhibitory neurotransmitter acting mainly in the brainstem and spinal cord. Nevertheless, in this work, no changes in glycine levels were observed in the brainstem. Glycine also acts as an excitatory modulator of the NMDA receptors [42]. The NMDA receptors are tetrameric complexes composed of obligatory NR1 subunits co assembled with different NR2 (A-D) and, less commonly, NR3 (A-B). Glutamate binds to the NR2 site while glycine binds to the NR1 site of NMDA receptors. After WBI, we found reduced levels of glycine in the hypothalamus. Previous studies reported that oral doses of glycine could improve sleep quality and reduce fatigue during the day, apparently through activation of NMDA receptors in the hypothalamic suprachiasmatic nucleus [43, 44]. Additionally, glycine agonists have anxiolytic and pro-cognitive effects and reduce brain injury induced by IL-1β [45, 46]. After an increase of IL-1β in the rat hypothalamus induced by the WBI [20], the decrease of glycine could be a damage mechanism induced by radiation that negatively influences the cellular homeostasis in the hypothalamus.

The reduction of taurine levels found in this study is consistent with previous endocrine hypothalamic effects also reported after WBI. Taurine is mainly produced by astrocytes and with a high concentration found in the hypothalamus [47]. Taurine microinjections in the hypothalamic arcuate nucleus can stimulate prolactin production in the pituitary gland [48]. Consistently with the reduced taurine levels, low prolactin concentrations have been reported after cranial irradiation in a rat model [37]. Furthermore, some children subjected to WBI have hypoprolactinemia, which have been correlated with lower growth hormone (GH) levels, a frequent sequel seen after treatment [49, 50]. Low prolactin levels could also affect stress response since prolactin has shown to reduce anxiety behavior, modulate neurogenesis, and exert neuroprotection [51]. Additionally, taurine may act as an anti-inflammatory and promote the cognitive function [52, 53]. Accordingly, lower taurine levels found in the hypothalamus could be contributing to endocrine and cognitive secondary effects reported after radiation treatments.

Aspartate levels were also reduced in the hypothalamus after radiation. Aspartate is an excitatory amino acid highly abundant in the hypothalamus [54]. Unlike glutamate, aspartate is a selective agonist of NMDA at the NR2 binding site. In the hypothalamus, aspartate and NMDA have been implicated in the regulation of hormonal release. Treatment with NMDA leads to enhanced prolactin and growth hormone secretion [54]. Therefore, similarly to taurine, low levels of aspartate could be implicated in endocrine disturbances observed after WBI.

WBI also modifies the prefrontal cortex inducing higher levels of the excitatory neurotransmitters glutamate and glutamine and a higher Gln/Glu ratio. The prefrontal cortex is known to be essential for higher cognitive functions. After brain irradiation, higher glutamate levels have been previously reported in other structures like the striatum [55]. Glutamate regulates synaptic transmission and plasticity by activating ionotropic (AMPA and NMDA) and metabotropic receptors (mGluR1-R8). Glutamate receptors overstimulation is known to induce potential damage in neural cells due to calcium overload [56]. Glutamine is transported from astrocytes into neurons where glutaminase deaminates glutamine to produce glutamate [57].

Consequently, an increased glutamine/glutamate ratio has been proposed to be associated with decreased glial function or dysfunction of glia–neuron communication [16]. Models of traumatic brain injury have also shown increases in glutamate and glutamine and imbalances in glutamate-glutamine/GABA [15]. In patients with schizophrenia, it has been reported that glutamine and glutamine/glutamate ratio is increased in the medial prefrontal cortex, which has been correlated with cognitive dysfunction [16]. Besides, glutamate increases in the prefrontal cortex of healthy patients have been correlated with cognitive and social dysfunctions [58]. In patients with diabetes type I, prefrontal glutamate-glutamine–gamma-aminobutyric acid (Glx) was increased and correlated with lower cognitive performance and mild depression [59]. These studies demonstrate that different pathophysiological conditions cause an imbalance of excitatory neurotransmission in the prefrontal cortex and the concurrent appearance of cognitive impairments. Interestingly, these two abnormalities can occur after WBI. Moreover, in this work, we also reported increased GABAa receptors in the prefrontal cortex after brain irradiation. Similar increases in prefrontal cortex GABAa receptors have been observed with aging [60]. Further, in hippocampal slices, irradiation increases the expression of GABAa, correlating with long-term potentiation (LTP) inhibition, which could be a mechanism involved in cognitive deficit [6].

Lastly, we reported that WBI increased the expression of the NR1 subunit of the NMDA receptor in the prefrontal cortex. Liang et al., using a higher dose of radiation (30 Gy), described the increased expression of NR1 and NR2A in the cortex, 1 and 2 months after irradiation [14]. Observed dissimilarities in NR2A could be associated with differences in dose escalation or coverage. The functional significance of the change in NR1 expression could be related to behavioral disturbances. The deletion of NR1 has been shown to stimulate social behavior [61]; by contrast, Iwata et al. demonstrated that radiation decreases the social interaction [62]. Thus, it seems plausible that WBI inhibits social behavior by increasing NR1 expression.

Care should be taken when extrapolating these results on radiation schemes other than WBI, as for brain Stereotactic Radiotherapy. The neurochemical effects of focused radiation may depend on the specific irradiated brain region and the received dose. For instance, in Stereotactic Radiotherapy, observed late behavioral effects are closely related to the dose spatial distribution and the dose received by specific regions [63]. Depending on dose distribution, even certain focused schemes can result in no behavioral changes [64]. Additionally, extreme increments to conventional dose rates, like experimental ultra-high dose rate schemes (flash), have been shown to reduce undesirable effects after whole-brain irradiation [27]. Therefore, future work can focus on evaluating regional neurochemical changes for different dose schemes.

## Conclusion

Results illustrate how WBI modifies differentially amino acid levels according to the analyzed brain region. At clinically equivalent doses, WBI can distinctively change neurotransmitters and receptors in the brainstem, hypothalamus, and the prefrontal cortex. At the hypothalamus, WBI decreases the concentration of inhibitory neurotransmitters and receptors while at the prefrontal cortex increase excitatory neurotransmitters and receptors. On the contrary, no changes were observed in the brainstem. Increments in Glutamate/GABA in the hypothalamus and Glutamine/Glutamate in the frontal cortex indicate modified neurochemical balance after irradiation.

We propose that observed changes could have an essential role in the etiology of the side effects after WBI and suggest new prospects for therapeutic targets. Hence, further studies should consider evaluations in both the hypothalamus and prefrontal cortex to better understand the involved mechanisms in radiotherapy-induced brain injury.

## Data Availability

The datasets supporting the conclusions of this article are included within the article and its additional files. They are also available from the corresponding author on reasonable request.

## Abbreviations

BED: Biologically effective dose
Fx: Fractions
RTOG: Radiation Therapy Oncology Group
HI: Homogeneity index defined as I_max_/RI, where, I_max_ = maximum isodose in the target, and RI = reference isodose
GABA: Gamma-aminobutyric acid
WBI: Whole-brain irradiation
NMDA: N-methyl-D-aspartate
Gly: Glycine
Tau: Taurine
Asp: Aspartate
Glu: Glutamate
Gln: Glutamine
RSS: Radiation somnolence syndrome
HPA: Hypothalamic–pituitary–adrenal
HPLC: High-performance liquid chromatography
OPA: Ortho-phthalaldehyde
RIPA: Radioimmunoprecipitation assay
BCA: Bicinchonic acid
TBST: Tris buffered saline plus 0.1% tween 20
IgG: Immunoglobulin G
HRP: Horseradish peroxidase
SPSS: Statistical package for the social sciences
GnRH: Gonadotropic releasing hormone
IL-1β: Interleukin 1 beta
MRS: Magnetic resonance spectroscopy
AMPA: α-Amino-3-hydroxy-5-methyl-4-isoxazolepropionic acid
Glx: Glutamate + glutamine + gamma-aminobutyric acid
LTP: Long-term potentiation
